# Dispositional Awe and Online Altruism: Testing a Moderated Mediating Model

**DOI:** 10.3389/fpsyg.2021.688591

**Published:** 2021-08-04

**Authors:** RongMao Lin, YanPing Chen, YiLin Shen, XiaXin Xiong, Nan Lin, Rong Lian

**Affiliations:** School of Psychology, Fujian Normal University, Fuzhou, China

**Keywords:** dispositional awe, online altruism, self-transcendent meaning in life, subjective socioeconomic status, moderated mediating model

## Abstract

Dispositional awe has a positive effect on prosociality. However, it has not been tested whether this disposition appears in online altruism. Using a large sample of 3,080 Chinese undergraduates, this study tested a moderated mediating model that takes self-transcendent meaning in life (STML) as a mediator and subjective socioeconomic status (SSES) as a moderator. As predicted, dispositional awe was positively correlated with online altruism, partly *via* the indirect effect of STML. SSES moderated both the direct and indirect effects. Specifically, the predictive effects of dispositional awe on both online prosocial behavior and STML were greater for lower rather than higher SSES. This study extends the prosociality of dispositional awe to cyberspace. Other implications are also discussed herein.

## Introduction

Dispositional awe and its prosociality have received increased attention recently. It refers to a disposition toward global awe, which arises from a perception of vastness and a need to accommodate the perception into existing mental schemas (Keltner and Haidt, 2003). Dispositional awe is a central emotional experience of religion, politics, nature, and art (Bonner, 2015). It also mixes respect with wonder, admiration, appreciation, and sometimes fear and anxiety (Schneider, 2017). Though it is complex and sometimes can be colored by appraisals of threat, it enjoys positive or self-transcendent character. It can broaden and build the mindset of individuals and resources (Stellar et al., 2017), enable them to gain a spiritual perspective on their life (Preston and Shin, 2016), and encourage people to transcend their own needs and desires (Jiang et al., 2018). Dispositional awe belongs to self-transcendent experience, but it differs from Cloninger's self-transcendence (1993), for it only involves the disposition or the tendency in the type of self-transcendent emotion rather than the comprehensive components of personality, temperament, and character (Cloninger, 1993).

The prosocial role of dispositional awe can be explained theoretically from the self-transcendent emotion perspective. Specifically, it can make self-decrease and motivate people to be good to others (Stellar et al., 2017; Keltner and Piff, 2020). An increasing number of studies have also found that awe promotes social connection and fosters prosocial behavior (Piff et al., 2015; Prade and Saroglou, 2016; Guan et al., 2019; Li et al., 2019). For example, Prade and Saroglou (2016) found that the induction of awe leads to increase prosocial behavioral intentions of generosity and help to the person in need. Similar results were showed in Guan et al. (2019) and Lin et al. (2020) studies, with a positive effect of dispositional awe on prosocial tendency measured by Prosocial Tendencies Measures (PTM). Further studies showed that awe fosters prosocial actions by reducing self-focus and diminishing self (e.g., Piff et al., 2015; Bai et al., 2017).

The positive effect of dispositional awe in prosocial actions has been confined to face-to-face interactions; however, whether and how it associates with online altruism have not yet been tested. As cyberspace plays an increasingly important part in modern life (Emond and West, 2003; Meredith, 2020), prosocial behavior also transfers to the Internet (Amichai-Hamburger, 2008; Sproull et al., 2013), and thus online altruism has become a salient form of prosociality. Compared with offline prosocial behavior, online altruism becomes more convenient and hidden and even comes into a new prosocial pattern (Amichai-Hamburger, 2008, 2013), for the Internet has sought to find a way that infinite informative resources can be combined and shared for the benefit of the public (Emond and West, 2003; Meredith, 2020). So far, whether people with higher levels of dispositional awe tend to detonate, help, or share in the net has not been clear. The main purpose of this study is to explore the relationship between dispositional awe and online altruism and its internal mediating mechanism and conditional process, extending evidence for the prosociality of dispositional awe from face-to-face to online interactions.

### Dispositional Awe and Online Altruism

Online altruism refers to voluntary actions intended to help or benefit another individual or group online (Wang and Wang, 2008). Given that cyberspace provides a unique space for people and significantly influences their cognition and behavior, online altruism likely manifests traits unique to those that are seen in face-to-face communications. Compared with offline interactions, online altruism happens more frequently and diversely as it is not constrained by time and physical space (Wright and Li, 2011; Sproull et al., 2013). Amichai-Hamburger (2008) proposed that online altruism manifests unique informative and communicative features at personal, interpersonal, and group levels. At the personal level, for example, the Internet can support volunteers with these advantages: ease of accessing information, freedom to search for information, access to the largest information resources in the world, and overcoming disabilities (Amichai-Hamburger, 2008). Online altruism often manifests in donating funds to worthy causes during online browsing of charity websites (Bennett, 2009), sharing information or successful experience with others *via* social software (Han et al., 2018), and online social behavior supporting people in need (Zhao and Basnyat, 2018). Furthermore, online altruism tends to be more hidden for the volunteers and recipients who do not need to contact each other face to face (Sproull et al., 2013).

Paralleled to its positive effect on face-to-face prosociality, dispositional awe may also be positively associated with online altruism. First, previous studies have offered sufficient evidence for the prosociality of dispositional awe in the offline world (Piff et al., 2015; Prade and Saroglou, 2016; Bai et al., 2017). From the perspective of the social function of self-transcendent emotion (Stellar et al., 2017), dispositional awe encourages people to transcend their momentary needs and desires, pay more attention to the requirements of others, embrace collaborative social groups, and engage in collective action. Furthermore, transforming the positive association from offline to online should be supported by the co-construction theory (Subrahmanyam et al., 2006; Wright and Li, 2011). It proposes that people tend to connect their offline worlds with online spaces, thereby generalizing their behavior from the face-to-face to the digital world. Targeting on the association between dispositional awe and prosociality, the co-construction theory also assumes that people with higher levels of dispositional awe generalize their altruistic traits to the digital world beyond face-to-face communications (Amichai-Hamburger, 2008; Wright and Li, 2011). Moreover, recent studies have also shown that techniques relating to digital space (such as virtual reality) are effective for inducing the emotion of awe (Chirico et al., 2016, 2017; Alice et al., 2018), indicating that awe may also manifest similar effect online relative to offline.

### The Indirect Effect of Self-Transcendent Meaning in Life

How dispositional awe positively associates with online altruism is as yet unclear. From the perspective of self-transcendence (Frankl, 1966; Wong, 2016; Stellar et al., 2017), this study further tests the indirect effect of self-transcendent meaning in life (STML) to answer this question. STML refers to a belief in transcending the individual living state in pursuit of a higher perspective (Li, 2006). People with higher levels of STML tend to believe that wins and losses in daily life are normal, dialectical, and meaningful. They hold a transcendent view and attitude toward life, and thus pay more attention to other groups, regardless of their interest (Le, 2010; Machell et al., 2015).

The self-transcendence theory of Frankl (1966) primordially explains the role of awe in the meaning of life. As a primary spiritual motivation and/or belief, self-transcendence seeks to express itself through our striving toward something greater than ourselves, and it represents our spiritual need to be connected with others and with a higher power, which also results in several self-transcendent feelings including awe, gratitude, appreciation, and peak experience (Frankl, 1966; Wong, 2016). Furthermore, the self-transcendent emotion theory (Stellar et al., 2017), which recently clarities a serious of positive emotions from their social function, can more precisely describe the relationship between dispositional awe and self-transcendent meaning of life. The self-transcendent emotion refers to a category of positive emotions, including awe, love, elevation, appreciation, etc., which is characterized as a vividly social function to bind individuals together in social relationships (Stellar et al., 2017). From the self-transcendent emotional perspective, dispositional awe, which is characterized in self-decreasing and increased feelings of connectedness, can urge people to transcend from the everyday concerns, beliefs, and actions of oneself toward a connection with a more meaningful, larger, and inclusive perspective of the world (Haidt and Morris, 2009; Stellar et al., 2017), and thus motivates individuals to engage in more collective and prosocial behavior. Moreover, the indirect effect role of STML was directly supported by the relationships between dispositional awe and a prosocial tendency (Li et al., 2019; Lin et al., 2020), so as subjective well-being (Zhao et al., 2019).

### The Moderating Effect of Subjective Socioeconomic Status

From the social-cognitive perspective of emotion, the question of whether the positive effect of dispositional awe on online altruism is influenced by subjective socioeconomic status (SSES) has yet to be explored. SSES is the perception of an individual for their socioeconomic standing, which is typically measured through self-reporting of the social class to which they belong (Demakakos et al., 2008; Kraus and Park, 2017). Due to its subjective character (Quon and McGrath, 2014), researchers have proposed that SSES influences the personality, emotion, and health of an individual more significantly, as compared to its objective counterpart (Nobles et al., 2013; Quon and McGrath, 2014; Bucciol et al., 2015).

The role of SSES can be described *via* the social class theory (Kraus et al., 2009, 2012; Kraus and Park, 2017). It proposes that different levels of social class significantly influence the sense of self, social perceptions, and related behavior of an individual. Specifically, given that diminished resources and a lower social rank constrain the social outcomes of individuals, people with lower levels of SSES show a more contextual orientation, with explaining social events from a relational social cognitive scheme and focusing on external, uncontrollable social forces, and other individuals who influence their life outcomes. On the other hand, those who enjoy abundant resources and an elevated rank create context and have higher levels of SSES, tend to be solipsistic in their social orientation, thereby explaining social events from a more self-oriented social cognitive scheme, and focusing on their own internal state, goals, motivation, and emotions (Kraus et al., 2012). Numerous studies have supported the significant moderation effect of SSES on personality, cognition, emotion, and psychological health (Maisel and Karney, 2012; Assari et al., 2018).

A lower level of SSES may benefit the positive effect of dispositional awe in online altruism. On the one hand, individuals with lower SSES tend to be more prosocial. From the social cognitive perspective, individuals with lower SSES are contextualists; they favor explanations of behavior that involve forces outside of individual control. They are more vigilant against threats, experience a reduced personal sense of control, develop more communal self-concepts, feel more compassion, and behave more prosocially (Kraus et al., 2012). On the other hand, individuals with lower SSES may have an increased tendency to experience awe. As the primordial model of awe describes, the emotion centers on the profound reaction of a subordinate to a powerful leader (Keltner and Haidt, 2003). Therefore, people with lower SSES are inclined to be awestruck by those in a higher social class. Empirical studies have supported the notion that lower SSES is positively associated with higher dispositional awe and benefits the positive effect of openness on dispositional awe (Lin et al., 2021).

### The Current Study

**Figure 1:**
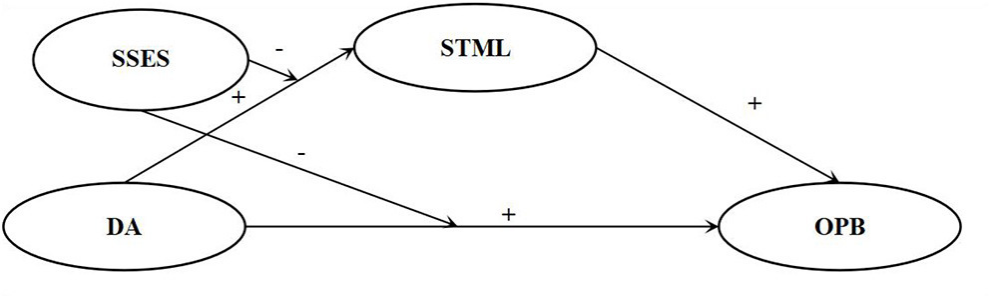
The hypothesized model. SSES, subjective socioeconomic status; DA, dispositional awe; STML, self-transcendent meaning in life; OPB, online prosocial behavior.

Thus far, the prosociality of dispositional awe has been tested only in face-to-face contexts, and not on the Internet. The main purpose of this study is to explore whether and how dispositional awe correlates with online altruism. In this study, a large sample of Chinese undergraduates was recruited. In line with the aforementioned studies, we hypothesized that: (1) dispositional awe would be positively correlated with online altruism (H1); (2) STML would show an indirect effect on the relationship between dispositional awe and online altruism (H2); and (3) SSES would moderate the direct relationship between dispositional awe and online altruism (H3) and the indirect effect of STML (H4 specifically, moderating the front path from dispositional awe to STML). The hypothesized model in this study is presented in Figure 1.

## Method

### Participants

A convenience-based cluster sampling of 3,080 Chinese undergraduates was recruited from Fujian Province in PRC. When surveyed, several kinds of universities including one normal university, two comprehensive universities, one medicinal university, and one science and technology university were covered, and balancing distributions in undergraduate major, gender, and grade were also considered. Of the total, 1,231 were men (40.0%) and 1,849 were women (60.0%); 819 were freshmen (26.6%), 946 were sophomores (30.7%), 830 were juniors (26.9%), and 485 were seniors (15.7%); the mean age of the participants was 19.9 years (*SD* = 1.4), and ages ranged from 18 to 26. The survey was approved by the Academic Ethics Committee of Fujian Normal University.

### Measures

#### Dispositional Awe

Dispositional awe was measured by the Dispositional Awe Questionnaire for Chinese Undergraduates (DAQ-CU) (Lin and Lian, 2020; Lin et al., 2020). The DAQ-CU includes 25 items consisting of 5 factors: awe of life (e.g., *I am often awestruck by life*), awe of nature (e.g., *I am often awestruck by nature*), awe in relationships (e.g., *I often feel the authority from my parents and teachers)*, awe in morality (e.g., *I often feel the binding force of law and/or rules*), and awe in spirit/religion (*e.g., I often feel the sanctity of religious rituals and/or activities*). All items are assessed on a six-point Likert scale ranging from 1 (*does not describe me at all*) to 6 (*describes me completely*), with higher scores indicating higher levels of dispositional awe. Previous studies have supported the high reliability and validity of the DAQ-CU in samples of Chinese undergraduates (Lin and Lian, 2020; Lin et al., 2020), and it has been widely used to measure dispositional awe in Confucian culture (e.g., Lin et al., 2020, 2021). In this study, the composite reliability (*ω*) of the DAQ-CU was 0.97, and its subscales ranged from 0.67 to 0.88; Cronbach's α coefficient for the DAQ-CU was 0.90, and its subscales ranged from 0.66 to 0.82. Confirmatory factor analysis (CFA) showed the five-factor model of DAQ-CU was acceptable: χ^2^ = 459.336 (*df* = 248), *p* < .001, root mean square error of approximation (*RMSEA*) (90% *CI*) = 0.056 [0.048, 0.064], comparative fit index (*CFI*) = 0.921, and Tucker–Lewis index (*TLI*) = 0.905.

#### Online Altruism

Online altruism was measured with the Internet Altruistic Behavior Scale (IABS), which was developed by Zheng et al. (2011). The IABS includes 26 items consisting of four factors: internet support (e.g., *Caring for and encouraging others on the Internet*), internet guidance (e.g., *Guiding others on how best to use the Internet*), internet sharing (e.g., *Sharing successful learning experiences with others on the Internet*), and internet reminding (e.g., *Reminding others about traps on the Internet*). The measure requires participants to respond on a four-point Likert scale ranging from 1 (*never or very rarely*) to 4 (*always or very often*), with higher scores indicating more online altruism. Previous studies have shown that IABS enjoys high reliability and validity in Chinese populations (Zheng et al., 2011, 2018). In this study, the composite reliability (ω) of the IABS was 0.96, and its subscales ranged from 0.84 to 0.89; Cronbach's α coefficient for the total IABS was 0.96, and its subscales ranged 0.85 to 0.96. CFA showed the four-factor model of IABS was acceptable: χ^2^ = 732.867 (*df* = 284), *p* < 0.001, *RMSEA* (90% *CI*) = 0.059 [0.054, 0.065], *CFI* = 0.914, and *TLI* = 0.902.

#### Self-Transcendent Meaning in Life

The STML was measured with the eight-item STML scale (SMLS), which assesses self-transcendent beliefs of individuals and understanding of meaning in life. It consists of two aspects: meaning obtained from failure (e.g., *Loss may be more meaningful than gain in life*), and self-transcending success and/or failure (e.g., *More success/failure more experience of life*). It is measured on a four-point Likert scale ranging from 1 (*disagree very much*) to 4 (*agree very much*), with higher scores indicating higher levels of self-transcendence in the meaning of life. Previous studies have shown Cronbach's α coefficient of 0.79, indicating that scores significantly correlate with mental health and thus support high reliability and validity (Li, 2002, 2006). In this study, the composite reliability (ω) of the SMLS was 0.88, and its subscales ranged from 0.74 to 0.82; Cronbach's α coefficient of the SMLS was 0.84, and its subscales ranged from 0.73 to 0.81. CFA showed the two-factor model of STML was acceptable: χ^2^ = 112.219 (*df* = 19), *p* < 0.001, *RMSEA* (90% *CI*) = 0.040 [0.033, 0.047], *CFI* = 0.979, *TLI* = 0.969.

#### Subjective Socioeconomic Status

Subjective socioeconomic status was measured by the Subjective Social Status Scale for Chinese Adolescents (SSSC-A), which was developed by Hu et al. (2012). The SSSC-A consists of two items that reflect the perceived family and school socioeconomic statuses of an individual. All the items are scored on a 10-point Likert scale ranging from 1 (*lowest*) to 10 (*highest*). The test–retest reliability for a 3-week interval was 0.78. The total score significantly correlates with depression and anxiety, with high reliability and validity. In this study, the composite reliability (ω) was 0.64, and Cronbach's α coefficient was 0.68.

### Statistical Analysis

All data were analyzed with SPSS version 25.0 and Mplus version 7.4 for Windows. Invalidated responses (blank or responses repeating the same option) were deleted listwise. Given that the data were collected *via* the self-report method, there was a risk of common method bias (Richardson et al., 2009; Podsakoff et al., 2012). Harman's single-factor test, based on exploratory factor analysis (EFA; along with unrotated principal component factor analysis), was performed first. Means, SDs, and Pearson's correlations among the studied variables were also reported in the primary analysis. Structural equation modeling (SEM) was employed to examine the hypothesized model. In the model, dispositional awe, online altruism, and STML were considered latent variables, and their measurement indicators were parceled using an isolated approach (Little et al., 2002). SSES was directly considered as a measuring variable with the mean total score, as it was measured with only two items. Bias-corrected bootstrapping (*N* = 1,000) method was employed to estimate CI of the indirect effect of STML (MacKinnon et al., 2004). The moderating effect was defined as the mean total SSES score multiplied by the mean total score for dispositional awe. The model was estimated by a robust maximum likelihood estimation procedure. Several fit statistics in the model evaluation were as follows: *TLI, CFI*, standardized root mean square residual (SRMR), and *RMSEA*. The following criteria were used to evaluate fit: *CFI* and *TLI* should be ≥0.90, and *RMSEA* and SRMR equal to or <0.08 (Marsh et al., 2004; Wen et al., 2004).

## Results

### Primary Analyses

The EFA showed that more than nine distinct factors with eigenvalues larger than 1.0 were retained. The first factor accounted for only 22.4% of the total variance, which is <40% (Richardson et al., 2009; Podsakoff et al., 2012). These results indicated that common method bias was not serious in this study.

Means, SDs, and Pearson's correlations for the study variables are presented in Table 1. There were significant gender differences for dispositional awe and online altruism, with women showing higher levels than men for dispositional awe [(4.726 ± 0.568) vs. (4.562 ± 0.660), *t* = 7.374, *p* < 0.001, *d* = 0.005], and vice versa for online altruism [(2.361 ± 0.629) vs. (2.197 ± 0.615), *t* = 7.163, *p* < 0.001, *d* = 0.005]. There were significant differences by grade for online altruism, with freshmen showing higher levels than students in other grades [(2.372 ± 0.640) vs. (2.230 ± 0.607), (2.207 ± 0.597), (2.239 ± 0.662), *F* = 11.798, *p* < 0.001, $\eta_{p}^{2}$ = 0.005].

**Table 1:** Means, SDs, and Pearson's correlations among studies variables (*n* = 3,080).

	**1**	**2**	**3**	**4**
1 Dispositional awe	–			
2 Online prosocial behavior	0.166^***^	–		
3 Self-transcendent meaning in life	0.312^***^	0.206^***^	–	
4 Subjective social statues	−0.054^**^	−0.110^***^	−0.064^***^	–
*M*	4.660	2.263	3.047	5.293
*SD*	0.612	0.626	0.458	1.312

^*^p < 0.05,

** *p < 0.01*,

*** *p < 0.001*.

Dispositional awe was positively correlated with online altruism, and STML; online altruism also positively correlated with STML. SSES was negatively associated with dispositional awe, online altruism, and STML (see Table 1).

### Hypothesized Model Test

A CFA was first performed to test the fit of the measurement model. The fit indices were as follows: χ^2^ = 1,670.643 (*df* = 88), *p* < 0.001, *RMSEA* (90% *CI* = 0.078 [0.075, 0.081], *CFI* = 0.911, and *TLI* = 0.894. Considering the sizeable sample in this study, the value of χ^2^ was large and significant; the value of *TLI* was close to 0.90, and the other fit indices attained the acceptable standards (Marsh et al., 2004; Wen et al., 2004). Thus, the measurement model was accepted, and a further hypothesized moderated mediating model needed to be tested.

**Figure 2:**
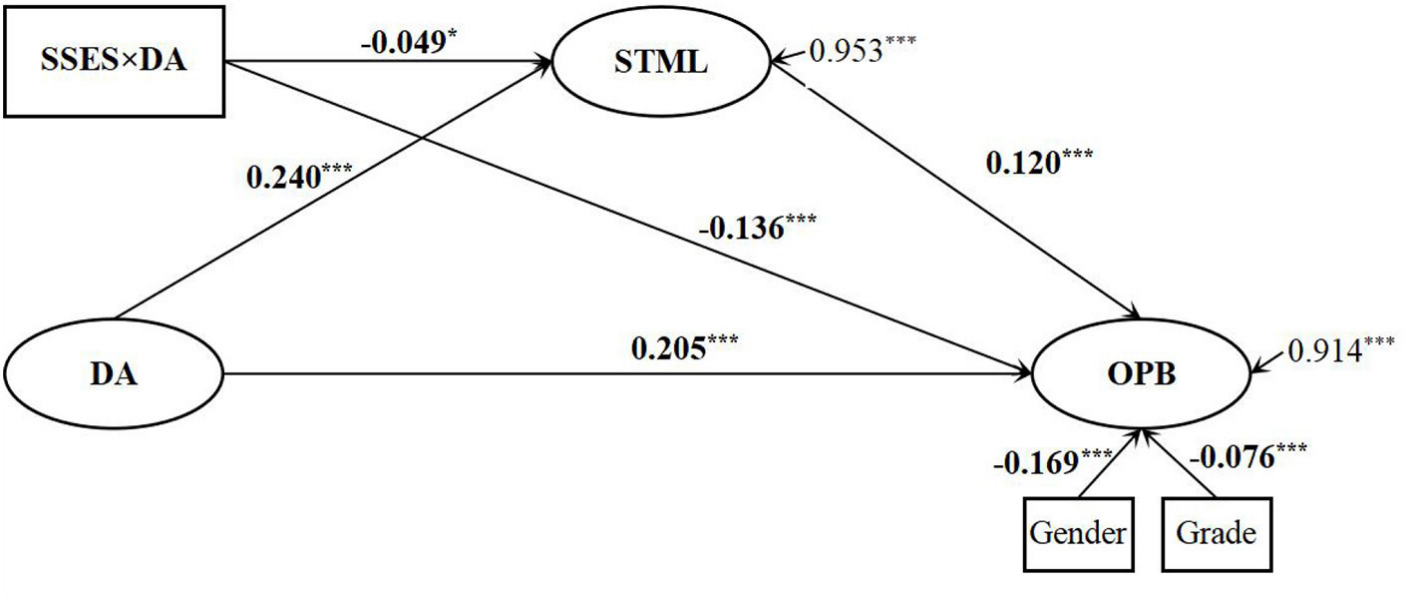
The standardized path coefficients of hypothesized model. SSES, subjective socioeconomic status; DA, dispositional awe; STML, self-transcendent meaning in life; OPB, online prosocial behavior. Note: *p < 0.05, **p < 0.01, ***p < 0.001.

The hypothesized model was initially constructed with gender and grade controlled. The results showed that it was nearly acceptable: [χ^2^ = 1415.037 (*df* = 81), *p* < 0.001, *RMSEA* (90% *CI*) = 0.075 [0.072, 0.078], *CFI* = 0.915, and *TLI* = 0.896]. After checking the path loading values, we found that the value of the path from SSES to online altruism was not significant (*β* = 0.159, *SE* = 0.177, *p* = 0.368). Modification should be conducted in the hypothesized model. With deleting the non-significant path, a modified moderated mediating model was further tested. The fit indices of the modified model were significantly improved and indicated that the modified moderated mediating model was acceptable: [χ^2^ = 1414.865 (*df* = 83), *p* < 0.001, *RMSEA* (90% *CI*) = 0.074 [0.070, 0.077], *CFI* = 0.924, and *TLI* = 0.907]. As shown in Figure 2, dispositional awe was positively correlated with online altruism, and thus the first hypothesis (H1) was supported. Dispositional awe was positively correlated with STML, and STML was positively correlated with online altruism. The standard indirect effect of STML was 0.029 (estimated = 0.052, *SE* = 0.011, *p* < 0.001, 95% *CI* ranging from 0.033 to 0.078). The second hypothesis (H2) was supported. For the total effect of dispositional awe on online altruism (ES = 0.029 + 0.205 = 0.234), the direct effect accounted for 87.6%, and the indirect effect of STML accounted for 12.4%.

**Figure 3:**
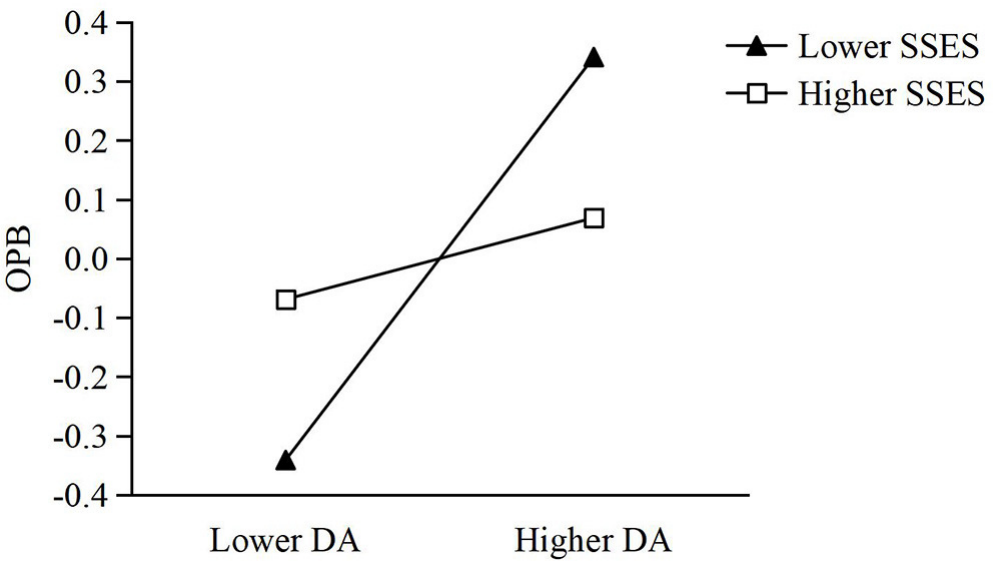
Moderation effect of SSES in the relationship between DA and OPB. SSES, subjective socioeconomic status; DA, dispositional awe; OPB, online prosocial behavior.

**Figure 4:**
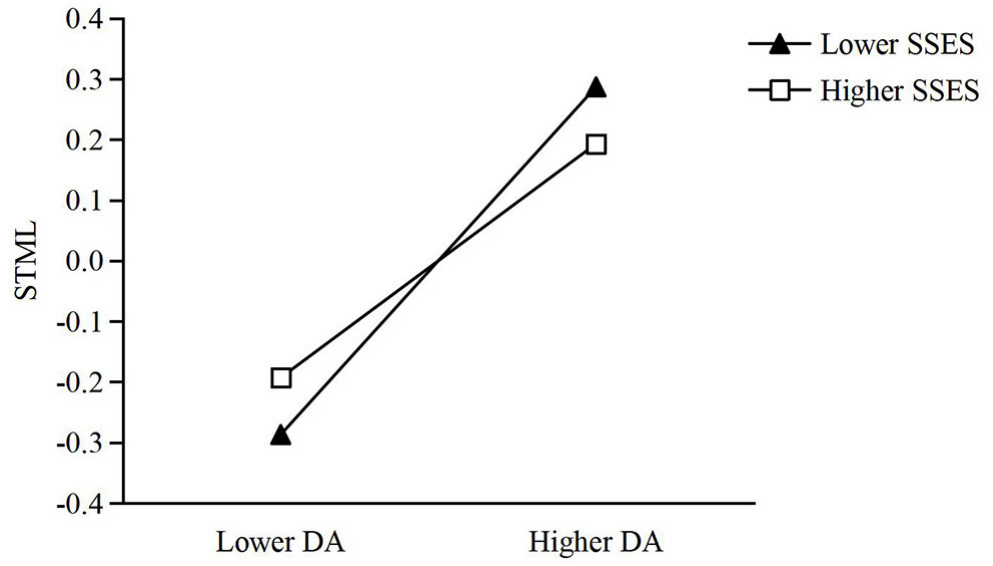
Moderation effect of SSES in the relationship between DA and STML. SSES, subjective socioeconomic status; DA, dispositional awe; STML, self-transcendent meaning in life.

The moderated mediating effect was also tested. As shown in Figure 2, the moderating effects of SSES on both online altruism and STML were negative (*β* = −0.136, −0.049), and the moderated mediating effect was also negative, with its standard effect size being −0.006 (estimate = −0.001, *SE* = 0.000, *p* = 0.041, 95% *CI* ranging from −0.001 to −0.001). The third and fourth hypotheses were also supported (H3 and H4). Then a simple slope was computed to further examine this moderating effect. As illustrated in Figures 3, 4, greater dispositional awe corresponded with higher levels of online altruism and STML for both low and high SSES, but the predictive effect of dispositional awe was greater for lower rather than higher SSES (0.341 vs. 0.069, 0.287 vs. 0.193, *p*_*s*_ < 0.001).

## Discussion

The main purpose of this study was to explore whether and how dispositional awe is associated with online altruism by testing a moderated mediating model in which STML was a mediator and SSES a moderator. The results show that dispositional awe was positively correlated with online altruism, which was in turn partially mediated by STML; SSES showed a moderating effect on this relationship. It moderated not only the direct correlation but also the indirect effect of STML (specifically, the front path from dispositional awe to STML). Therefore, the hypothesized moderated mediating model in this study is supported. This study extends the prosociality of dispositional awe from offline to online and describes that dispositional awe has a positive effect on online altruism.

First, this study found that dispositional awe is positively associated with online altruism. This study extends the prosociality of dispositional awe, whether in face-to-face communication or *via* the digital world. As a self-transcendent emotion, dispositional awe can lead people to transcend their current frame of reference and trigger a relative diminishment of self, thereby increasing prosocial behavior (Piff et al., 2015; Perlin and Li, 2020). The prosociality of dispositional awe is supported by the co-construction theory (Subrahmanyam et al., 2006; Wright and Li, 2011). Although it offers more convenience, diversity, and covertness (Sproull et al., 2013), online altruism is identical to what occurs offline to a certain extent, in that the altruism trait is beneficial to other people and/or social groups (Carlo and Randall, 2002; Penner et al., 2004). People with higher levels of dispositional awe will also generalize their prosocial behavior to the digital world extending beyond face-to-face communications.

Second, this study demonstrates the indirect effect of STML, partially explaining how dispositional awe positively correlates with online altruism. This result is consistent with previous studies targeting subjective well-being (Zhao et al., 2019). Whether face to face or online, people with higher dispositional awe pay reduced attention to self-oriented concerns (Chen and Mongrain, 2020). They hold a transcendent view and attitude toward life and go beyond their own momentary needs, thereby paying more attention to other-oriented concerns and engaging in more collective and prosocial behavior (Piff et al., 2015; Prade and Saroglou, 2016). The path from dispositional awe to STML demonstrates a motivational process for self-transcendence on prosocial behavior, for the dispositional awe as a special self-transcendent emotion will motivate people's self-transcendent belief and view (Van Cappellen et al., 2013), leading them to lay down their own desire and demand (Van Cappellen and Rimé, 2014), considering more about the other and group's need, and thus making sacrifices to engage in prosocial behavior in the net. Considering the cross-sectional data in the study, there may be an alternative explanation for the correlation between dispositional awe and STML. From the perspective of the self-transcendence theory (Frankl, 1966; Wong, 2016), self-transcendent belief in the meaning of life triggers the awe in turn, which then increases higher levels of online altruism. This may be a cognitive process for the self-transcendence on prosocial behavior. To some extent, no matter the motivational or cognitive process, this study enriches the self-transcendent theory with a new explanation for online altruism.

Furthermore, this study supports the moderating effect of SSES, specifying the social condition necessary for the positive effect of dispositional awe in online altruism. The results show that compared with higher levels of SSES, individuals with lower SSES may benefit from the positive effect of dispositional awe in both online altruism and the indirect effect of STML (see Figures 3, 4). The beneficial effect of lower SSES should be explained from a contextual orientation, based on social class theory (Kraus et al., 2009, 2012; Kraus and Park, 2017). Generally, people with lower levels of SSES recognize social events from a contextual social cognitive scheme and focus on the external and uncontrollable social forces impacting their lives, for they perceive themselves as having diminished and limited social resources and a lower social rank. Lower levels of SSES benefiting the positive effect of dispositional awe serve as evidence that primordial awe centers upon the emotional reaction of a subordinate to a powerful leader again (Keltner and Haidt, 2003). People with lower levels of SSES are not only inclined to be awestruck by people with higher socioeconomic status (relative to themselves), they also develop more communal self-concepts, pay more attention to others than to themselves, and thus behave more prosocially.

In sum, this study extends the prosociality of dispositional awe from face-to-face to online communication through testing *via* a moderated mediating model. This study is significant for both the theoretical study of awe and the applied research of cyber-psychology. This research was able to determine how and when dispositional awe is positively correlated with online altruism, further extending the cyber-condition of the prosociality of awe. This study expands the condition of prosociality of awe and further clarifies the psychological mechanism in the positive effect of prosocial behavior from the self-transcendent emotion perspective. This study also contributes to some extent to the positive psychology, providing more evidence for the self-transcendent theory. Furthermore, it is significant in practice with the background of multinetwork culture. Moral decline and relating problems in the net have become a prominent social topic. This study suggested motivational progress targeting at these problems. Especially, cultivating the awe of online users will be an effective path to increase online prosocial and altruistic behavior and create a more positive Internet environment.

This study has several limitations. First, it only measured awe on the level of individual common propensity and did not specifically consider this disposition on the Internet and further test the emotional state during specific activities. Further study should consider the specificity of awe on the Internet, and especially explore the state of awe when people in the net, further investigating whether and how online awe influences online group behavior. Second, considering that the direct effect of dispositional awe was still large when the indirect effect of STML was comprehended, warranting the other weighted internal variables in the relationship between dispositional awe and online altruism. Future studies should explore the intermediate variable basing on the special Internet, further finding out more and closer indirect effects in the online awe and altruism. Moreover, this study conducted a self-reporting method and cross-sectional design, which resulted in common method bias and did not determine which is cause or result. Future work should apply a longitudinal approach to further explore the positive effect of dispositional awe and the mediating effect of STML.

## Data Availability Statement

The raw data supporting the conclusions of this article will be made available by the authors, without undue reservation.

## Ethics Statement

The studies involving human participants were reviewed and approved by Academic Committee of Fujian Normal University. The patients/participants provided their written informed consent to participate in this study.

## Author Contributions

RML conceived and designed this study, collected and analyzed the data, written, and revised the manuscript. RML, YPC, YLS, XXX, and NL revised the manuscript. RL and RML were responsible for the study. All authors contributed to the article and approved the submitted version.

## Conflict of Interest

The authors declare that the research was conducted in the absence of any commercial or financial relationships that could be construed as a potential conflict of interest.

## Publisher's Note

All claims expressed in this article are solely those of the authors and do not necessarily represent those of their affiliated organizations, or those of the publisher, the editors and the reviewers. Any product that may be evaluated in this article, or claim that may be made by its manufacturer, is not guaranteed or endorsed by the publisher.

## Appendix

### Dispositional Awe Questionnaire for Chinese Undergraduates

1. I am often awestruck by nature.

2. I often experience peace and harmony from nature.

3. I struggle to pursue more value in my limited life.

4. I often admire the greatness of life.

5. I am often shocked by the creativity of nature.

6. I feel extremely small in front of nature.

7. I respect the fairness of rules.

8. I often feel the binding force of law and/or rules.

9. I believe Big Brother is watching over head.

10. It is a wisdom to hold morals in awe.

11. I easily get close to and integrate into nature.

12. I think law and/or rules cannot be violated.

13. I often feel the seriousness of morality, law and/or rules.

14. I think the awe for life is an optimistic attitude.

15. I want to cherish the life for it is given but once.

16. I often feel a sense of distance from my parents and teachers.

17. I often feel the authority from my parents and teachers.

18. I often sigh for the fragility of life.

19. I often feel the sanctity of religious rituals and/or activities.

20. I want to get close to my parents and teachers, but I'm a little afraid of them.

21. I am full of awe for my parents and teachers.

22. I often praise the value of freedom.

23. I often experience the sanctity of belief.

24. I am full of gratitude for my parents and teachers.

25. I often feel that temples, churches, and/or statues are mysterious.

## References

[B1] Alice C. Francesco F. Lorenzo C. Andrea G. (2018). Designing awe in virtual reality: an experimental study. Front. Psychol. 8:2351. 10.3389/fpsyg.2017.02351 29403409 PMC5786556

[B2] Amichai-Hamburger Y. (2008). Potential and promise of online volunteering. Comput. Human Behav. 24, 544–562. 10.1016/j.chb.2007.02.004

[B3] Amichai-Hamburger Y. (2013). The Social Net: Understanding Our Online Behavior. Oxford: Oxford University Press. 10.1093/acprof:oso/9780199639540.001.0001

[B4] Assari S. Preiser B. Lankarani M. M. Caldwell C. H. (2018). Subjective socioeconomic status moderates the association between discrimination and depression in African American youth. Brain Sci. 8, 71–84. 10.3390/brainsci8040071 29677115 PMC5924407

[B5] Bai Y. Maruskin L. A. Chen S. Gordon A. M. Stellar J. E. McNeil G. D.. (2017). Awe, the diminished self, and collective engagement: universals and cultural variations in the small self. J. Pers. Soc. Psychol. 113, 185–209. 10.1037/pspa0000087 28481617

[B6] Bennett R. (2009). Impulsive donation decisions during online browsing of charity websites. J. Consumer Behav. 8, 116–134. 10.1002/cb.277

[B7] Bonner E. (2015). Exploring Dispositional Awe and Its Relationship with Spiritual Intelligence: Measuring Dispositional Awe as a Multidimensional Construct. (Doctor), Northcentral University Graduate Faculty of the School of Behavioral and Health Sciences, Prescott Valley, Arizona.

[B8] Bucciol A. Cavasso B. Zarri L. (2015). Social status and personality traits. J. Econ. Psychol. 51, 245–260. 10.1016/j.joep.2015.10.002

[B9] Carlo G. Randall B. A. (2002). The development of a measure of prosocial behaviors for late adolescents. J. Youth Adoles. 31, 31–44. 10.1023/A:1014033032440

[B10] Chen S. K. Mongrain M. (2020). Awe and the interconnected self. J. Positive Psychol. 10.1080/17439760.2020.1818808. [Epub ahead of print].

[B11] Chirico A. Cipresso P. Yaden D. B. Biassoni F. Riva G. Gaggioli A. (2017). Effectiveness of immersive videos in inducing awe: an experimental study. Sci. Rep. 7, 1218–1229. 10.1038/s41598-017-01242-0 28450730 PMC5430774

[B12] Chirico A. Yaden D. B. Riva G. Gaggioli A. (2016). The potential of virtual reality for the investigation of awe. Front. Psychol. 7:1766. 10.3389/fpsyg.2016.01766 27881970 PMC5101419

[B13] Cloninger C. R. (1993). A psychobiological model of temperament and character. Arch. Gen. Psychiatry 50, 975–990. 10.1001/archpsyc.1993.01820240059008 8250684

[B14] Demakakos P. Nazroo J. Breeze E. Marmot M. (2008). Socioeconomic status and health: the role of subjective social status. Soc. Sci. Med. 67, 330–340. 10.1016/j.socscimed.2008.03.038 18440111 PMC2547480

[B15] Emond B. West R. L. (2003). Cyberpsychology: a human-interaction perspective based on cognitive modeling. Cyberpsychol. Behav. 6, 527–536. 10.1089/109493103769710550 14583128

[B16] Frankl V. (1966). Self-transcendence as a human phenomenon. J. Humanistic Psychol. 6, 97–106. 10.1177/002216786600600201

[B17] Guan F. Chen J. Chen O. Liu L. Zha Y. (2019). Awe and prosocial tendency. Curr. Psychol. 38, 1033–1041. 10.1007/s12144-019-00244-7

[B18] Haidt J. Morris J. P. (2009). Finding the self in self-transcendent emotions. Proc. Nat. Acad. Sci. U.S.A. 106, 7687–7688. 10.1073/pnas.0903076106 PMC2683117 19416850

[B19] Han J.-T. Chen Q. Liu J.-G. Luo X.-L. Fan W. (2018). The persuasion of borrowers' voluntary information in peer to peer lending: an empirical study based on elaboration likelihood model. Comput. Human Behav. 78, 200–214. 10.1016/j.chb.2017.09.004

[B20] Hu M.-l. Wang M.-C. Cai L. Zhu X.-Z. Yao S.-Q. (2012). Development of subjective socioeconomic status scale for chinese adolescents. Chin. J. Clin. Psychol. 20, 155–161.

[B21] Jiang L. Yin J. Mei D. Zhu H. Zhou X. (2018). Awe weakens the desire for money. J. Pacific Rim Psychol. 12, 1–10. 10.1017/prp.2017.27

[B22] Keltner D. Haidt J. (2003). Approaching awe, a moral, spiritual, and aesthetic emotion. Cogn. Emot. 17, 297–314. 10.1080/02699930302297 29715721

[B23] Keltner D. Piff P. K. (2020). Self-transcendent awe as a moral grounding of wisdom. Psychol. Inq. 31, 160–163. 10.1080/1047840X.2020.1750927

[B24] Kraus M. W. Park J. W. (2017). The structural dynamics of social class. Curr. Opin. Psychol. 18, 55–60. 10.1016/j.copsyc.2017.07.029 28830036

[B25] Kraus M. W. Piff P. K. Keltner D. (2009). Social class, sense of control, and social explanation. J. Pers. Soc. Psychol. 97, 992–1004. 10.1037/a0016357 19968415

[B26] Kraus M. W. Piff P. K. Mendoza-Denton R. Rheinschmidt M. L. Keltner D. (2012). Social class, solipsism, and contextualism: how the rich are different from the poor. Psychol. Rev. 119, 546–572. 10.1037/a0028756 22775498

[B27] Le T. N. (2010). Life satisfaction, openness value, self-transcendence, and wisdom. J. Happiness Stud. 12, 171–182. 10.1007/s10902-010-9182-1

[B28] Li H. (2002). College Stress and Psychological Well-being: Vision in Life as a Coping Resource. University of HongKong.

[B29] Li H. (2006). Self-transcendence meaning of life moderates in the relation between college stress and psychological well-being. Acta Psychol. Sinica 38, 422–427. 10.1097/00024382-200610001-00089

[B30] Li J.-J. Dou K. Wang Y.-J. Nie Y.-G. (2019). Why awe promotes prosocial behaviors? The mediating effects of future time perspective and self-transcendence meaning of life. Front. Psychol. 10:1140. 10.3389/fpsyg.2019.01140 31191387 PMC6548882

[B31] Lin R.-M. Hong Y.-J. Xiao H.-W. Lian R. (2020). Dispositional awe as self-transcendent experience: the mediating role of self-transcendence in prosocial tendency. Soc. Behav. Pers. 48, 1–10. 10.2224/sbp.9665

[B32] Lin R.-M. Lian R. (2020). The characters of dispositional awe in Chinese undergraduates. J. Jimei Univ. 21, 64–70.

[B33] Lin R. M. Hong Y. J. Xiao H.-W. Chen Y.-P. Lian R. (2021). Openness to experience and dispositional awe: the moderating role of subjective socioeconomic status and mediating role of Zhong-Yong thinking style in Chinese undergraduates. Scand. J. Psychol. 62, 617–624. 10.1111/sjop.12728 34036581

[B34] Little T. D. Cunningham W. A. Shahar G. Widaman K. F. (2002). To parcel or not to parcel: exploring the question, weighing the merits. Struct. Equat. Model. A Multidiscipl. J. 9, 151–173. 10.1207/S15328007SEM0902_1

[B35] Machell K. A. Kashdan T. B. Short J. L. Nezlek J. B. (2015). Relationships between meaning in life, social and achievement events, and positive and negative affect in daily life. J. Pers. 83, 287–298. 10.1111/jopy.12103 24749860

[B36] MacKinnon D. P. Lockwood C. M. Williams J. (2004). Confidence limits for the indirect effect: distribution of the product and resampling methods. Multivariate Behav. Res. 39, 99–128. 10.1207/s15327906mbr3901_4 20157642 PMC2821115

[B37] Maisel N. C. Karney B. R. (2012). Socioeconomic status moderates associations among stressful events, mental health, and relationship satisfaction. J. Family Psychol. 26, 654–660. 10.1037/a0028901 22686266

[B38] Marsh H. Hau K.-T. Wen Z. (2004). In search of golden rules: comment on hypothesis-testing approaches to setting cutoff values for fit indexes and dangers in overgeneralizing Hu and Bentler's (1999) findings. Struct. Equ. Model. 11, 320–341. 10.1207/s15328007sem1103_2

[B39] Meredith J. (2020). Conversation analysis, cyberpsychology and online interaction. Soc. Personal. Psychol. Compass 14, 285–294. 10.1111/spc3.12529

[B40] Nobles J. Weintraub M. R. Adler N. E. (2013). Subjective socioeconomic status and health: relationships reconsidered. Soc. Sci. Med. 82, 58–66. 10.1016/j.socscimed.2013.01.021 23453318 PMC4171062

[B41] Penner L. A. Dovidio J. F. Piliavin J. A. Schroeder D. A. (2004). Prosocial behavior: multilevel perspectives. Annu. Rev. Psychol. 56, 365–392. 10.1146/annurev.psych.56.091103.070141 15709940

[B42] Perlin J. D. Li L. (2020). Why does awe have prosocial effects? New perspectives on awe and the small self. Perspect. Psychol. Sci. 15, 291–308. 10.1177/1745691619886006 31930954

[B43] Piff P. K. Dietze P. Feinberg M. Stancato D. M. Keltner D. (2015). Awe, the small self, and prosocial behavior. J. Pers. Soc. Psychol. 108, 883–899. 10.1037/pspi0000018 25984788

[B44] Podsakoff P. M. MacKenzie S. B. Podsakoff N. P. (2012). Sources of method bias in social science research and recommendations on how to control it. Annu. Rev. Psychol. 63, 539–569. 10.1146/annurev-psych-120710-100452 21838546

[B45] Prade C. Saroglou V. (2016). Awe's effects on generosity and helping. J. Positive Psychol. 11, 522–530. 10.1080/17439760.2015.1127992

[B46] Preston J. L. Shin F. (2016). Spiritual experiences evoke awe through the small self in both religious and non-religious individuals. J. Exp. Soc. Psychol. 70, 212–221. 10.1016/j.jesp.2016.11.006

[B47] Quon E. C. McGrath J. J. (2014). Subjective socioeconomic status and adolescent health: a meta-analysis. Health Psychol. 33, 433–447. 10.1037/a0033716 24245837 PMC5756083

[B48] Richardson H. A. Simmering M. J. Sturman M. C. (2009). A tale of three perspectives examining post hoc statistical techniques for detection and correction of common method variance. Organ. Res. Methods 12, 762–800. 10.1177/1094428109332834

[B49] Schneider K. (2017). The resurgence of awe in psychology: promise,hope, and perils. Humanistic Psychol. 45, 103–108. 10.1037/hum0000060

[B50] Sproull L. Conley C. Moon J. Y. (2013). Prosocial behavior on the net, in The Social Net: Understanding Human Behavior in Cyberspace, eds Amichai-Hamburger Y. (Oxford: Oxford University Press), 139–161.

[B51] Stellar J. E. Gordon A. M. Piff P. K. Cordaro D. Anderson C. L. Bai Y.. (2017). Self-transcendent emotions and their social functions: compassion, gratitude, and awe bind us to others through prosociality. Emot. Rev. 9, 200–207. 10.1177/1754073916684557

[B52] Subrahmanyam K. Smahel D. Greenfield P. M. (2006). Connecting developmental processes to the Internet: identity presentation and sexual exploration in online teen chatrooms. Dev. Psychol. 42, 395–406. 10.1037/0012-1649.42.3.395 16756432

[B53] Van Cappellen P. Rimé B. (2014). Positive emotions and self-transcendence, in Religion, Personality, and Social Behavior, ed Saroglou V. (New York, NY: Psychology Press), 123–145. 10.4324/9780203125359-13

[B54] Van Cappellen P. Saroglou V. Iweins C. Piovesana M. Fredrickson B. L. (2013). Self-transcendent positive emotions increase spirituality through basic world assumptions. Cogn. Emot. 27, 1378–1394. 10.1080/02699931.2013.787395 23662802

[B55] Wang C.-C. Wang C.-H. (2008). Helping others in online games: prosocial behavior in cyberspace. Cyberpsychol. Behav. 11, 344–346. 10.1089/cpb.2007.0045 18537505

[B56] Wen Z. L. Hau K.-T. Marsh H. W. (2004). Structural equation model testing: cutoff criteria for goodness of fit indices and chi-square test. Acta Psychol. Sinica 36, 186–194. 10.1007/BF02911031

[B57] Wong P. T. P. (2016). Meaning-seeking, self-transcendence, and well-being, in Logotherapy and Existential Analysis: Proceedings of the Viktor Frankl Institute, Vol. 1, ed Batthyany A. (Cham: Springer), 311–322. 10.1007/978-3-319-29424-7_27

[B58] Wright M. F. Li Y. (2011). The associations between young adults' face-to-face prosocial behaviors and their online altruisms. Comput. Human Behav. 27, 1959–1962. 10.1016/j.chb.2011.04.019

[B59] Zhao H. Zhang H. Xu Y. He W. Lu J. (2019). Why are people high in dispositional awe happier? The roles of meaning in life and materialism. Front. Psychol. 10:1208. 10.3389/fpsyg.2019.01208 31191402 PMC6540826

[B60] Zhao X. Basnyat I. (2018). Online social support for “Danqin Mama”: a case study of parenting discussion forum for unwed single mothers in China. Comput. Human Behav. 80, 12–21. 10.1016/j.chb.2017.10.045

[B61] Zheng X. Xie F. Ding L. (2018). Mediating role of self-concordance on the relationship between internet altruistic behaviour and subjective wellbeing. J. Pacific Rim Psychol. 12, e1–e7. 10.1017/prp.2017.14

[B62] Zheng X. L. Zhu C. L. Gu H. G. (2011). Development of internet altruistic behavior scale for college students. Chinese J. Clin. Psychol. 19, 606–608. 10.1111/j.1600-0714.2011.01024.x

